# Port site recurrence of esophageal adenocarcinoma after minimally invasive esophagectomy: a case report

**DOI:** 10.1186/s40792-020-00861-6

**Published:** 2020-05-11

**Authors:** Taichi Horino, Yoshifumi Baba, Daichi Nomoto, Kazuto Harada, Yukiharu Hiyoshi, Yohei Nagai, Masaaki Iwatsuki, Shiro Iwagami, Yuji Miyamoto, Naoya Yoshida, Hideo Baba

**Affiliations:** Department of Gastroenterological Surgery, Graduate School of Medical Sciences, Kumamoto University, 1-1-1 Honjo, Kumamoto, 860-8556 Japan

**Keywords:** Port site recurrence, Esophageal cancer, Adenocarcinoma, Minimally invasive esophagectomy

## Abstract

**Background:**

Port site recurrence has been observed after a variety of oncologic resection procedures. However, few have reported port site recurrence of esophageal cancer.

**Case presentation:**

A 51-year-old man underwent minimally invasive esophagectomy for pT3(AD)N3M0 adenocarcinoma of the esophagus. One year after surgery, he presented with a rapidly growing tumor on the right thoracic wall. Contrast computed tomography demonstrated an enhancing tumor with uptake on positron emission tomography. We performed resection of the thoracic wall, including the skin and subcutis. The pathologic diagnosis was poorly differentiated adenocarcinoma, consistent with metastasis of esophageal origin.

**Conclusion:**

This was the first report on thoracic port site recurrence of esophageal adenocarcinoma. We recommend elimination of leakage around the thoracoscopic ports to prevent such recurrence. We should provide prudent postoperative clinical surveillance.

## Introduction

Port site recurrence was first reported by Dobronte et al. in 1978 and has been observed after a variety of oncologic resection procedures [1]. However, few have reported port site recurrence of esophageal cancer. Herein, we described the case of thoracic port site recurrence 1 year after minimally invasive esophagectomy for poorly differentiated esophageal adenocarcinoma.

## Case presentation

A 51-year-old man presented with dysphagia. Barium swallow showed an irregular stricture in the middle to lower thoracic esophagus. Endoscopy showed a mucosal nodularity that was located 28 cm from the incisors and a tumor stricture that encompassed the area between 32 and 42 cm from the incisors. Computed tomography (CT) revealed lower thoracic paraesophageal lymph node metastasis.

The patient underwent minimally invasive esophagectomy using two 5-mm ports and three 12-mm ports. We placed the 5-mm ports on the third intercostal anterior axillary line and the eight intercostal posterior axillary line. The 12-mm ports were placed on the fifth and seventh intercostal anterior axillary line and the ninth intercostal middle axillary line. The patient was kept in the left semi-prone position, and we used 8–10 mmHg carbon dioxide gas to create pneumothorax during the procedure. The postoperative course was uneventful, except for abdominal wall scar hernia that was repaired on the 15th postoperative day. The tumor was 12 cm in size, and the resected specimen was margin-negative. Pathologic examination confirmed poorly differentiated adenocarcinoma infiltrating the adventitial layer of the esophagus and lower thoracic paraesophageal lymph node metastasis [pT3(AD)N3M0, p Stage III AJCC/UICC 8th Ed.].

**Fig. 1:**
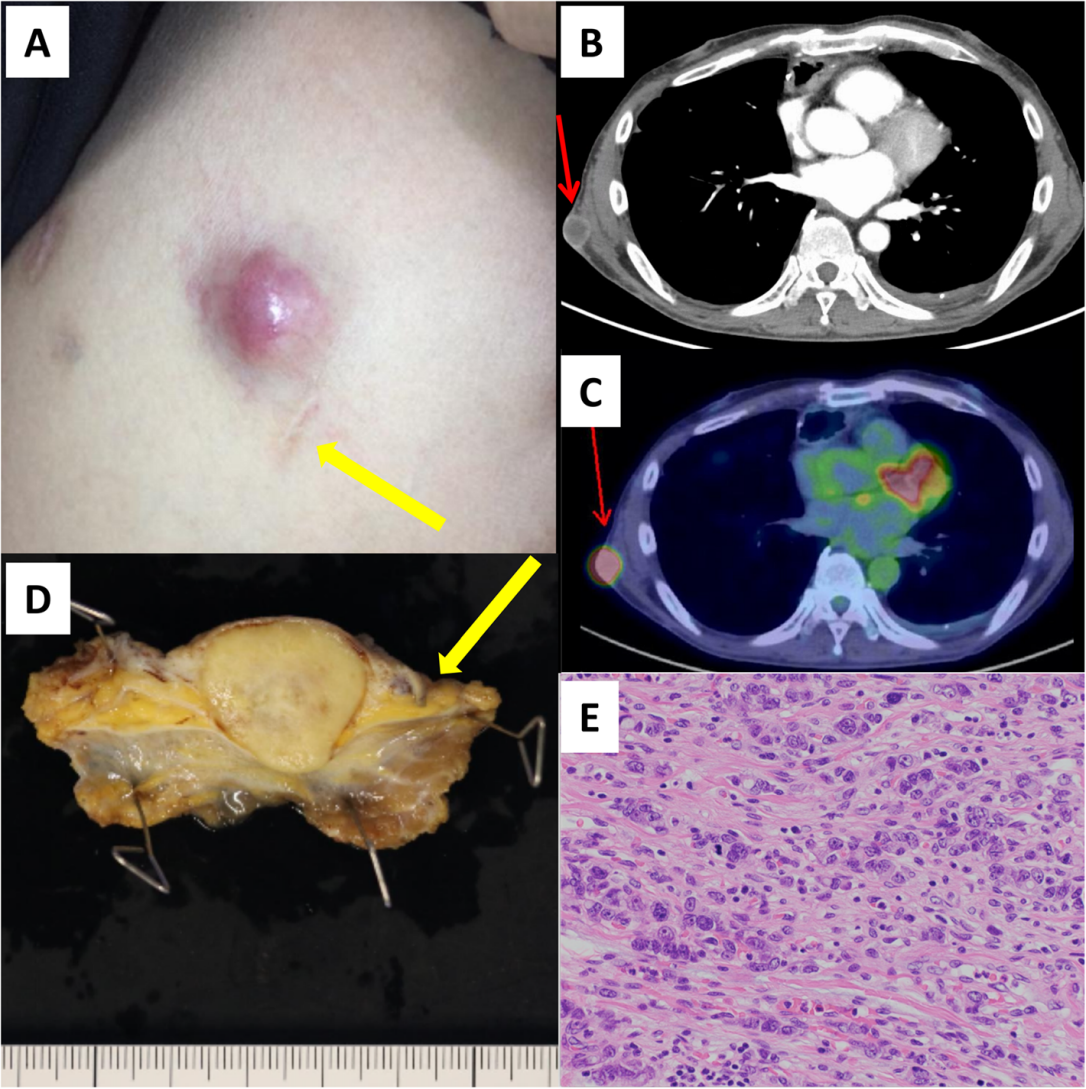
**a** On the thoracic wall, there is an erythematous tumor that has the size of a thumb tip and is mobile. **b** Contrast CT demonstrates an enhancing tumor that is separated from the ribs. **c** PET-CT shows uptake in the tumor site. **d** Macroscopic image of the resected tumor. **e** Histopathologic image of recurrent tumor (hematoxylin and eosin stain). Yellow arrow shows the scar of the 5-mm surgical port site. Red arrow shows the recurrent lesion

One year after surgery, he presented with a rapidly growing tumor on the right thoracic wall. On physical examination, the tumor had the size of a thumb tip and was erythematous and mobile; it was located between the sixth and seventh ribs just above the scar of the 5-mm surgical port site (Fig. 1a). Contrast CT demonstrated an enhancing tumor that was separated from the ribs (Fig. 1b). Positron emission tomography-CT showed uptake in the tumor site (Fig. 1c). No other signs of metastasis or recurrence were found by imaging.

We performed resection of the thoracic wall, including the skin and subcutis. Macroscopically, the 22-mm tumor was solid and contained a scirrhous area (Fig. 1d). Histologic examination of the specimen showed restiform proliferation of atypical cells with intracellular mucus (Fig. 1e). There was necrotic change in the core of the tumor. The diagnosis was poorly differentiated adenocarcinoma, consistent with metastasis of esophageal origin.

Based on the absence of imaging evidence of recurrence in other sites and the negative margin on pathologic examination after esophagectomy, we considered this case as local recurrence, which was completely removed by thoracic wall resection. He was simply followed up without adjuvant chemotherapy and is under regular surveillance.

## Discussion

Recently, thoracoscopic and laparoscopic procedures have been spreading as the methods of oncologic resection worldwide. Generally, port site recurrence is rare, and most reports on this condition were after cholecystectomy or colorectal surgery [2, 3]. Recent studies reported approximately 1% incidence of port site recurrence [3].

The incidence of esophageal adenocarcinoma is relatively rare in Japan, approximately 6.5–7.1% of all esophageal carcinomas [4]. At present, the primary treatment of esophageal carcinomas has been surgery. Minimally invasive esophagectomy was first described in 1990s. The procedure has been widely spread because it has the potential advantages of being a less traumatic procedure than open esophagectomy [5]. However, in English language literature, we could find only five cases of port site recurrence after esophagectomy [6–8]. Table 1 summarizes the clinical features of the five previously published cases, including this report, of port site recurrence of esophageal carcinoma after esophagectomy. As shown in the table, three cases of port site recurrence of esophageal squamous cell carcinoma have already been reported [6, 7]. Siegal et al. reported a case of laparoscopic port site recurrence of adenocarcinoma after esophagectomy [8]. However, to the best of our knowledge, this was the first report on thoracic port site recurrence of esophageal adenocarcinoma. The reason why port site recurrence is rare after minimally invasive esophagectomy for esophageal adenocarcinoma remains unknown. We acknowledge that further experiences are necessary to confirm the etiology of port site recurrence of esophageal adenocarcinoma.

**Table 1:** Literature review of cases with port site recurrence of esophageal carcinoma after esophagectomy

Source	Age	Sex	Pathologic findings	Period until recurrence	Treatment for recurrence	Outcome
Dixit et al. (1999) [6]	72	F	T2N0M0 SCC	6 months	None	No data
Yamamoto et al. (2009) [7]	50	M	T2N1M1a SCC	3 months	Radiotherapy	4 months (died of pleuritis carcinomatosa)
Yamamoto et al. (2009) [7]	59	M	T3N1M0 SCC	4 months	Thoracic wall resection	8 months (died of pleuritis carcinomatosa)
Yamamoto et al. (2009) [7]	59	M	T4N1M1a SCC	6 months	Radiotherapy	20 months (died of pleuritis carcinomatosa)
Siegal et al. (2017) [8]	62	M	T1bN0M0 adenocarcinoma	2 months	External beam electron therapy	No data
Present case	51	M	T3N0M0 adenocarcinoma	12 months	Thoracic wall resection	1 month (alive)

*SCC* squamous cell carcinoma

There are some theories on the etiology of port site metastasis after endoscopic surgery. Hubens et al. advocated the “chimney effect” theory, which suggested that the high pressure gradient created by pneumoperitoneum can result in subsequent outflow of floating tumor cells through the port wound, thereby leading to metastasis [3, 9]. Although this theory was said to be unexpected in a thoracotomy wound [7], it can theoretically happen in any high thoracic pressure condition, such as pneumothorax. We hypothesized the etiology to be secondary to the outflow of tumor cells and fluid leak that can lead to implantation of malignant cells. In this case, the recurrence site was located immediately above the scar of the 5-mm port, which we did not use for handling tumor samples. Although such leakage is difficult to prevent during operation, it should be minimized to reduce the risk for port site recurrence.

The indications for adjuvant therapy in cases of port site recurrence depend on the presence of other sites of recurrence or dissemination. Yamamoto et al. provided radiotherapy to the pleural cavity that showed signs of dissemination on CT [7]. On the other hand, Siegal et al. reported the case of a patient who underwent palliative external beam electron therapy that was decided on after a multidisciplinary discussion [8]. In our case, we simply followed up the patient after thoracic wall resection without adjuvant treatment, because we considered it as local recurrence. We will certainly continue careful surveillance of the patient.

## Conclusion

Thoracoscopic port site recurrence after minimally invasive esophagectomy can occur. We recommend elimination of leakage around the thoracoscopic ports to prevent such recurrence. In addition, the risks for port site recurrence should be recognized and prudent postoperative clinical surveillance should be provided.

## Data Availability

All data generated or analyzed during this study are included in this published article.

## Abbreviations

CT: Computed tomography
PET: Positron emission tomography
AJCC: American Joint Committee on Cancer
UICC: Union for International Cancer Control
SCC: Squamous cell carcinoma
